# Resolving widespread and endemic dinoflagellates (Symbiodiniaceae) mutualistic with Indo‐Pacific octocorals reveals differences in specificity based on host phylogeny

**DOI:** 10.1111/jpy.70127

**Published:** 2026-02-22

**Authors:** Caleb C. Butler, Kira E. Turnham, Andy Hess, Todd C. LaJeunesse

**Affiliations:** ^1^ Department of Biology The Pennsylvania State University University Park Pennsylvania USA; ^2^ Cooperative Institute of Marine and Atmospheric Research NOAA Honolulu Hawaii USA; ^3^ Institute of Energy and the Environment The Pennsylvania State University University Park Pennsylvania USA

**Keywords:** biogeography, *Cladocopium*, co‐evolution, ecological specialization, horizontal transmission, new species, Octocorallia, soft corals

## Abstract

Endosymbionts in the dinoflagellate family Symbiodiniaceae can form mutualisms with a diverse array of host invertebrates, constituting a widespread and ecologically important family. While those associated with reef‐building corals (order Scleractinia) have received considerable research attention, the diversity and ecology of zooxanthellae from soft coral hosts (Octocorallia) have remained understudied and unappreciated. To address this lack of understanding, octocoral zooxanthellae were sampled across the Indo‐Pacific and genetic, morphological, ecological, and geographic evidence were utilized to formally characterize five new species in the genus *Cladocopium*. Four species were associated with hosts in the family Sarcophytidae that horizontally acquire their endosymbionts. Of these new species, *C. fabriciae* sp. nov. and *C. peratum* sp. nov. are widespread across the Indo‐Pacific whereas *C. zanzibariense* sp. nov. and *C. belauense* sp. nov. are known only from their type localities in Zanzibar and Palau, respectively. The fifth species, *C. bilineaum* sp. nov., occurs in the Pacific and Indian Oceans associated with hosts in the families Xeniidae, which can display either horizontal or vertical mode of transmission and Lemnaliidae, which horizontally transmit their endosymbionts. Because soft coral abundances are increasing with ocean warming across many geographic provinces and in various reef habitats, formal species descriptions of their endosymbionts should facilitate future physiological and ecological research toward a more comprehensive understanding of their natural history and contributions to coral reef ecosystem productivity.

**Abbreviations**
bp: base pairs
CI: confidence interval
*co*b: mitochondrial cytochrome b
*cox*1: mitochondrial cytochrome oxidase 1
DMSO: dimethylsulfoxide
EDTA: ethylenediaminetetraacetic acid
GBR: Great Barrier Reef
ITS2: internally transcribed spacer region 2
LSU: large ribosomal subunit
*mtMutS* or *msh*
: mitochondrial mismatch repair gene
MYA: million years ago
PAUP: phylogenetic analysis using parsimony
PCR‐DGGE: polymerase chain reaction – denaturing gel gradient electrophoresis
*psb*A^ncr^
: *psb*A minicircle non‐coding region

## INTRODUCTION

Many animals in the phylum Cnidaria form obligate mutualisms with endosymbiotic dinoflagellates (i.e., zooxanthellae). During the establishment of these partnerships, symbionts are obtained from the environment (horizontal transmission) or inherited directly from the parent either via oogenesis or brooding during embryogenesis (vertical transmission). Indo‐Pacific reef‐building corals (order Scleractinia) that vertically transmit symbionts during oogenesis associate with highly specific host species (Turnham et al., 2021). In contrast, taxa that acquire symbionts horizontally from the environment tend to associate with host‐generalist symbionts (Butler et al., 2023). In scleractinians, the mode of symbiont acquisition influences the ecology and evolution of these symbioses and explains the lack of overlap in symbiont fidelity between vertical and horizontal transmitters (LaJeunesse et al., 2010; Lewis et al., 2024). Whether patterns of host‐symbiont specificity also relate to the host's mode of symbiont acquisition displayed by other cnidarian hosts remains largely unexplored.

Due to their importance in constructing and supporting coral reef ecosystems, studies on animal‐dinoflagellate mutualisms have focused mostly on hermatypic (reef building) scleractinians (class Hexacorallia). Indeed, progress in symbiont species taxonomy is biased for those associated with stony hexacorallians (Butler et al., 2023; LaJeunesse et al., 2014; Lewis et al., 2019; Turnham et al., 2021; Wham et al., 2017). Octocorals (class Octocorallia), however, are also dominant constituents of Indo‐Pacific and Atlantic reef systems and can reach densities up to 100 colonies · m^−2^ in Caribbean “octocoral forests” (Lasker et al., 2020, 2025). Many “soft corals” are more resilient to changing ocean conditions than stony coral populations, as indicated by their increasing abundances in recent decades (Fabricius, 1997; Johns et al., 2014; Kurihara et al., 2021; Tkachenko et al., 2022; Wakeford et al., 2008). Only a few studies have investigated dinoflagellate‐octocoral mutualisms over limited geographic areas (van Oppen et al., 2005) and often only at one location (Goulet & Coffroth, 2003b; Kirk et al., 2009; Liberman et al., 2022; Pelosi et al., 2021; Sammarco & Strychar, 2013; van Oppen et al., 2005).

Regional differences in octocoral‐symbiont partnerships exist: *Cladocopium* and *Durusdinium* dominate Indo‐Pacific hosts whereas Atlantic octocorals are dominated by *Breviolum* (van Oppen et al., 2005). Notably, genetically distinct lineages (i.e., species) of *Breviolum* in the Caribbean form highly specific mutualisms with host gorgonians (Goulet & Coffroth, 2003a; Kirk et al., 2005, 2009; Prada et al., 2014; Santos et al., 2003, 2004; Wham & LaJeunesse, 2016). Furthermore, studies of Red Sea octocorals have shown that the mode of symbiont acquisition corresponded to differences in host‐symbiont fidelity, similar to stony corals; hosts reliant on vertical transmission (by oogenesis) associated with *Symbiodinium* spp. (formerly Clade A) whereas those with horizontal transmission associated with *Cladocopium* spp. and *Durusdinium* spp. (formerly Clades C and D, respectively; Barneah et al., 2004; Liberman et al., 2022). These characterizations of symbiont diversity indicate a high degree of specificity between octocorals and their zooxanthellae, but much remains unknown about these Indo‐Pacific mutualisms where the host diversity is highest.

Most information on the identity and distribution of symbionts associated with Indo‐Pacific octocorals arises from studies broadly surveying host communities living in diverse reef habitats. These initial genetic comparisons (i.e., using the internal transcribed spacer, or ITS, rDNA region) revealed octocorals possess symbionts in the widespread genus *Cladocopium* and associate with specific lineages, or genetic “types,” that differ from those observed in reef‐building corals and other host taxa (LaJeunesse et al., 2003, 2004, 2010). The finding of genetically distinct lineages associated only with octocorals emphasizes the need for further research to better characterize their ecology and evolutionary history, which begins with establishing a formal taxonomy.

This study characterized species of *Cladocopium* associated with three ecologically abundant families of octocoral hosts in the sub‐class Malacalcyonacea—Sarcophytidae, Xeniidae, and Lemnaliidae—collected from various reefs across the Indo‐Pacific. Genetic, morphological, ecological, and biogeographic evidence was combined to resolve species associated with these octocorals. Additionally, the newly recognized octocoral symbionts were compared phylogenetically with taxa associated with scleractinians to examine the effect of divergent host lineages on patterns of symbiont diversification. Because of the widespread and increasing abundance of zooxanthellate Indo‐Pacific octocorals, more investigations into their physiology, biogeography, and ecology are needed. Formal species characterizations of their zooxanthellae should help promote future research of these important mutualisms.

## MATERIALS AND METHODS

### Field collections

Octocoral specimens were collected by SCUBA diving with scissors from reef sites across the Great Barrier Reef (GBR) of Australia, Palau, Taiwan, Zanzibar of Tanzania, and the Andaman Sea of western Thailand. Photographs of each colony sampled in Australia, Palau, and Tanzania were taken using various cameras. The samples used here were collected from 2002–2022 and included samples from published cnidarian‐Symbiodiniaceae biodiversity surveys (Table S1; LaJeunesse et al., 2003, 2004, 2010). Samples collected ranged from 1 to 3 cm^2^ and were preserved in a 20% dimethylsulfoxide (DSMO)/0.25 M ethylenediaminetetraacetic (EDTA)/sodium chloride (NaCl) saturated aqueous buffer (Seutin et al., 1991) or 95% ethanol at −20°C.

### DNA extraction, amplification, and sequencing

A modified Promega Wizard protocol was used to extract genomic DNA from 0.5 cm^2^ of each sample as described in LaJeunesse et al. (2003). The symbionts in many samples were characterized genetically, initially using polymerase chain reaction–denaturing gradient gel electrophoresis (PCR–DGGE) profiling of the nuclear internal transcribed spacer 2 (ITS2) rDNA region of the ribosomal array (LaJeunesse, 2002; LaJeunesse et al., 2004). Amplified products were examined with gel electrophoresis with a CB Scientific system (CBS Scientific, San Diego, CA, United States) with 45%–80% denaturing gradient gels. Band profiles (i.e., “fingerprints”) of each sample were visualized using SYBR Green stain and compared to lanes with known standards. The brightest individual bands were sequenced to confirm or characterize the symbiont's ITS2 “type” identity. Samples with *Cladocopium* were characterized further using additional DNA markers (Table S2), including the nuclear large ribosomal subunit rDNA geneLSU rDNA; ~600 base pairs or bp; Zardoya et al., 1995), mitochondrial cytochrome b gene (*co*b; ~900 bp), mitochondrial cytochrome oxidase 1 gene (*cox*1; 950 bp; Zhang et al., 2008), partial chloroplast 23S rDNA gene (cp23S; ~600 bp; Zhang et al., 2000), and the non‐coding region of the *psb*A minicircle (*psb*A^ncr^; ~900–1600 bp; Moore et al., 2003). The mitochondrial mismatch repair gene (*mtMutS* or *msh*1; ~850 bp) was used for host octocoral identification and phylogenetic reconstruction (Frances & Hoover, 2002; Sánchez & McFadden, 2003). Although this marker is not always able to discriminate species, it effectively discriminates numerous lineages within the family (especially genera within family Sarcophytidae; McFadden et al., 2011) and is consistent with the recent systematic revisions of class Octocorallia (see McFadden et al., 2022).

Successfully amplified products were cleaned and Sanger sequenced using the BigDye Terminator 3.1 Cycle Sequencing Kit (ThermoFisher Scientific, Waltham, MA, United States), with sequence reactions analyzed at the Penn State University Genomics Core Facility on an Applied Biosciences Sequencer. Only the *psb*A^ncr^ marker was sequenced in both directions due to its length. Base calling on chromatograms was visually inspected for accuracy in Geneious v 11.0.5 (Biomatters, Auckland, New Zealand). Default parameters in Geneious were used for pairwise alignment of the *psb*A^ncr^ marker.

### Phylogenetic analysis

Independent *Cladocopium* phylogenies were generated using both maximum parsimony and maximum likelihood approaches in PAUP (v4.0a169; Swofford, 2014) for each marker (i.e., LSU rDNA, *co*b, *cox*1, and cp23S genes). Initially, separate phylogenies were constructed for each gene marker to verify congruence among gene trees for both models (data not shown). Sequences were aligned using ClustalOmega (EMBL‐EBI). In PAUP, gaps and insertions were treated as fifth character states and scored as single changes. Branch support was assessed using 1000 bootstrap replicates. MrBayes v3.2.1 was used to assess posterior probabilities of nodes, implementing the general time reversible substitution model with gaps treated as missing data. Markov chain Monte Carlo simulations were run for a total of 1,000,000 generations, sampling every 100 generations. The initial 25% of sampled trees were discarded as burn‐in corresponding with the convergence of chains.

Because marker‐specific phylogenies utilizing both models showed consistent branching patterns, sequences from all conserved markers were concatenated to differentiate evolutionarily isolated lineages (LaJeunesse et al., 2015, 2022) and were compared against previously described *Cladocopium* (Butler et al., 2023; Hume et al., 2015; Lee et al., 2020; Trench & Blank, 1987; Turnham et al., 2021). The concatenated dataset was aligned using ClustalOmega (EMBL‐EBI), and PAUP (v4.0a169; Swofford, 2014) was again employed to generate the final maximum likelihood phylogeny utilizing a heuristic search strategy with *Halluxium pauxillum* as the outgroup (Nitschke et al., 2020).

Sequences of the non‐coding region of the dinoflagellate *psb*A minicircle (psb*A* ^ncr^), a rapidly evolving locus (LaJeunesse & Thornhill, 2011; Moore et al., 2003), were analyzed separately to further investigate lineage delineation and assess intra‐ and interspecific diversity, using the same methodologies used for the more conserved DNA markers. Proposed species were delineated based on support at deep nodes at any clade with 95% node support in maximum likelihood and Bayesian methodologies.

### Cell imaging and size measurements

Preserved dinoflagellate cells were viewed under bright‐field illumination at a total magnification of 400× (40× objective and 10× ocular) with an Olympus BX61 compound microscope (Olympus Corp, Tokyo, Japan) and imaged using the ORCA ER (Model C4742‐80) and Olympus DP71 at the Huck Institutes of the Life Sciences Microscopy Facility. The average lengths and widths of at least 150 cells were recorded per sample measured, selecting for diverse hosts and regions (if possible) for each putative symbiont species, and mean sizes and 95% confidence intervals (CI) were analyzed using R Statistical Software (v4.1.2; R Core Team, 2021, https://www.R‐project.org). Cell images for species description were taken at 100× on a Keyence Digital Microscope Model BZ‐9000E (Keyence, Osaka, Japan) under bright‐field oil immersion. Images were postedited for light levels and contrast in Photoshop (v.26.5; Adobe Systems, San Jose, CA, United States).

## RESULTS

### Species descriptions

Holotypes have been deposited in the Algal Collection of the National Herbarium, Smithsonian Institution, Washington DC, United States; herbarium acronyms follow Index Herbariorum online (Thiers, 2025).

### *Cladocopium fabriciae* C.C.Butler & LaJeunesse sp. nov. (Figure 2b)

Holotype: US 241300, June 2009, symbiont cells in tissues of *Sclerophytum* sp. preserved with 20% DMSO/0.25 M EDTA/NaCl saturated water, *leg*. Todd C. LaJeunesse

Type locality: Off the coast of the Maanshan Nuclear Power Plant in Hengchun Township, Taiwan (21°57.19′ N, 120°45.28′ E)

Description: Vegetative ovate cells range in mean length between 9.66 μm (±0.28 μm; 95% CI) and 10.93 μm (± 0.23 μm; 95% CI) and range in mean width between 8.43 μm (±0.27 μm; 95% CI) and 9.87 μm (±0.25 μm; 95% CI). Nucleotide sequences of the large subunit rDNA gene (GenBank PV873144), partial chloroplast large subunit gene (cp23s, GenBank PV873139), mitochondrial cytochrome b gene, (*co*b, GenBank PV868206), mitochondrial cytochrome oxidase gene, (*cox*1f, GenBank PV868211), and the full sequence of the *psb*A^ncr^ marker from the minicircle encoding the D1 protein of the photosynthesis reaction center II (GenBank PV868238–PV868241) genetically define this species.

Habitat: Common to many horizontally transmitting soft corals in the region of the Western Pacific. Particularly dominant on the Great Barrier Reef, but also observed in Palau, Taiwan, and rarely the reefs of Phuket in Thailand. Associates only with members of the family Sarcophytidae.

Etymology: Named after Dr. Katharina Fabricius in honor of her contributions to the field of octocoral biology.

Comment: Species were previously referred to in the literature as ITS2 “type” C1 but are not to be confused with other species whose ribosomal arrays are numerically dominated by the same “C1” ITS2 rDNA region sequence variant such as *Cladocopium goreaui*, *C. pacificum*, *C. latusorum*, *C. proliferum*, and *C. vulgare*.

### *Cladocopium peratum* C.C.Butler & LaJeunesse sp. nov. (Figure 2d)

Holotype: US 241299, April 2007, symbiont cells in tissues of *Sclerophytum* sp. preserved with 20% DMSO/0.25 M EDTA/NaCl saturated water, *leg*. Todd C. LaJeunesse

Type locality: Changuu in Zanzibar, Tanzania (6°7.21′ S, 39°10.13′ E)

Description: Vegetative ovate cells range in mean length between 9.65 μm (±0.20 μm; 95% CI) and 10.56 μm (±0.24 μm; 95% CI) and range in mean width between 8.21 μm (±0.21 μm; 95% CI) and 9.20 μm (±0.27 μm; 95% CI). Nucleotide sequences of the large subunit rDNA gene (GenBank PV873145), partial chloroplast large subunit gene (cp23s, GenBank PV873140), mitochondrial cytochrome b gene (*co*b, GenBank PV868207), mitochondrial cytochrome oxidase gene, (*cox*1 GenBank PV868212), and the full sequence of the *psb*A^ncr^ marker from the minicircle encoding the D1 protein of the photosynthesis reaction center II (GenBank PV868234–PV868237) genetically define this species.

Habitat: Common to horizontally transmitting soft corals in Zanzibar of Tanzania and the Great Barrier Reef of Australian of the Indo‐Pacific. Only known to associate with members of the family Sarcophytidae.

Etymology; Named after the Greek *peratos* for “on the opposite side” in reference to the unusual range of *Cladocopium peratum* on opposite sides of the Indian Ocean.

Comment: Species previously referred to in the literature as ITS2 “type” C65. Authors caution those utilizing the ITS2 rDNA region for species identification as one other “C65” lineage was uncovered, albeit in one sample (Figure 2a).

### *Cladocopium belauense* C.C.Butler & LaJeunesse sp. nov. (Figure 2e)

Holotype: US 241297, February 2009, symbiont cells in tissues of *Sarcophyton* sp. preserved with 20% DMSO/0.25 M EDTA/NaCl saturated water, *leg*. by Todd C. LaJeunesse

Type locality: Rebotel Reef in Palau (western Micronesia; 7°15.76′ N, 134°31.15′ E)

Description: Vegetative ovate cells range in mean length between 10.73 μm (±0.19 μm; 95% CI) and 11.61 μm (±0.19 μm; 95% CI) and range in mean width between 9.62 μm (±0.21 μm; 95% CI) and 10.53 μm (±0.19 μm; 95% CI). Nucleotide sequences of the large ribosomal subunit rDNA gene (GenBank PV873148), partial chloroplast large subunit gene (cp23s, GenBank PV873141), mitochondrial cytochrome b gene (*co*b, GenBank PV868208), mitochondrial cytochrome oxidase gene (*cox*1, GenBank PV868215), and the full sequence of the *psb*A^ncr^ marker from the minicircle encoding the D1 protein of the photosynthesis reaction center II (GenBank PV868242**–**PV868246) genetically define this species.

Habitat: Locally dominates the soft coral communities of Palau in the Pacific Ocean. One study purported observing a “C71” in the South China Sea associated with *Sarcophyton glaucum* (Gong et al., 2018), indicating that it has a broader regional distribution, but this report requires verification with other markers due to ITS2 rDNA region data being misleading for species identification, especially so in the C1 radiation, and thus may represent a different but closely related lineage.

Etymology: From the indigenous name for Palau, “Belau,” due to the symbiont first being identified in the reefs of Palau

Comment: Species previously referred to in the literature ITS2 “type” C71.

### *Cladocopium zanzibariense* C.C.Butler & LaJeunesse sp. nov. (Figure 2c)

Holotype: US 241296, April 2007, symbiont cells in tissues of *Sarcophyton* sp. preserved with 20% DMSO/0.25 M EDTA/NaCl saturated water, *leg*. Todd C. LaJeunesse

Type locality: Banda Kuu in Zanzibar, Tanzania (5°43.00′ S, 39°17.87′ E)

Description: Vegetative ovate cells range in mean length between 9.63 μm (±0.21 μm; 95% CI) and 10.46 μm (±0.23 μm; 95% CI) and range in mean width between 8.14 μm (±0.22 μm; 95% CI) and 9.18 μm (±0.25 μm; 95% CI). Nucleotide sequences of the large ribosomal subunit rDNA gene (GenBank PV873146), partial chloroplast large subunit gene (cp23s, GenBank PV873143), mitochondrial cytochrome b gene (*co*b, GenBank PV868209), mitochondrial cytochrome oxidase gene (*cox*1, GenBank PV868213), and the full sequence of the *psb*A^ncr^ marker from the minicircle encoding the D1 protein of the photosynthesis reaction center II (GenBank PV868231–PV868233) genetically define this species.

Habitat: Locally dominates the soft coral communities of Zanzibar in the Indian Ocean

Etymology: The species epithet is derived from the Latinization of “Zanzibar” where this species appears to be endemic.

Comment: Species previously referred to in the literature as ITS2 “type” C107ab

### *Cladocopium bilineaum* C.C.Butler & LaJeunesse sp. nov. (Figure 3b)

Holotype: US 241298, Aprl 2007, symbiont cells in tissues of *Xenia* sp. preserved with 20% DMSO/0.25 M EDTA/NaCl saturated water, *leg*. Todd C. LaJeunesse

Type locality: Changuu in Zanzibar, Tanzania (6°7.21′ S, 39°10.13′ E)

Description: Vegetative ovate cells range in mean length between 10.18 μm (±0.20 μm; 95% CI) and 13.30 μm (±0.40 μm; 95% CI) and range in mean width between 8.67 μm (±0.26 μm; 95% CI) and 10.89 μm (±0.33 μm; 95% CI). Nucleotide sequences of the large ribosomal subunit rDNA gene (GenBank PV873145), partial chloroplast large subunit gene (cp23s, GenBank PV873142), mitochondrial cytochrome b gene (*co*b, GenBank PV868210), mitochondrial cytochrome oxidase gene (*cox*1, GenBank PV868214), and the full sequence of the *psb*A^ncr^ marker from the minicircle encoding the D1 protein of the photosynthesis reaction center II (GenBank PV868216–PV868230) genetically define this species.

Habitat: Common to vertically transmitting corals Xeniidae (*Caementabunda*, *Xenia*, and other taxonomically undefined lineages), and the horizontally transmitting *Heteroxenia* and Lemnaliidae (*Lemnalia*, *Paralemnalia*, and *Rhytisma*) and can be observed across the Indo‐Pacific, spanning Zanzibar of Tanzania, the Great Barrier Reef of Australia, and the reefs of Palau, and has been identified in the Red Sea by Liberman et al. (2024)

Etymology: From the Latin “bi” for two and “linea” for line to denote its dual transmission modes and two lineages of closely related hosts

Comment: Species previously referred to in the literature as ITS2 “type” C64 and C64a.

### Zooxanthellae phylogenetics

**FIGURE 1:**
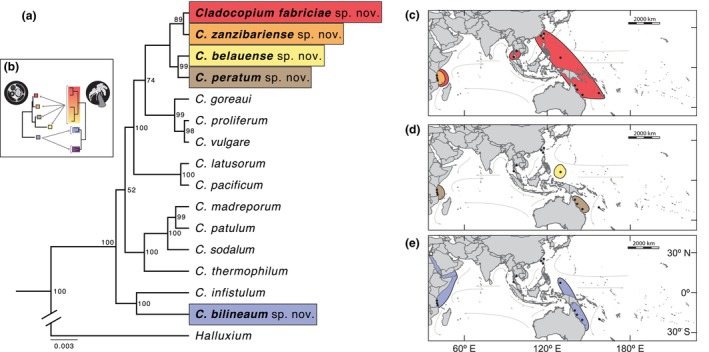
(a) Phylogenetic reconstruction of the genus *Cladocopium* inferred from aligned, concatenated DNA sequences of the large subunit ribosomal rDNA (LSU rDNA), partial chloroplast *cp23*, mitochondrial *co*b, and mitochondrial *cox*1 genes, showing the relationship of the newly described octocoral‐associated *Cladocopium* to the previously described species. Numerals at branch nodes represent the percentage bootstrap support values for maximum likelihood analysis. *Inset* (b): Comparison between the phylogenies of the octocoral symbiont (left) and associated octocoral host taxa (right). Color highlights on the host phylogeny corresponds to different octocoral families, from top to bottom: Sarcophytidae (red‐orange), Xeniidae (blue), and Lemnaliidae (purple). (c) Known distributions of *C. fabriciae* and *C. zanzibariense*. (d) Known distributions of *C. belauense* and *C. peratum*. (e) Distribution (from this study) of *C. bilineaum*. Dots on maps correspond to sample sites. The white square represents samples from Liberman et al. (2024) indicating the presence of *C. bilineaum* in the Gulf of Aqaba.

Independent phylogenetic reconstructions from DNA sequences (LSU rDNA, *co*b, *cox*1, and cp23s genes) were congruent, producing similar branching orders and clustering samples into putative species lineages (analyses not shown). The LSU rDNA and cp23s genes were conserved and did not resolve all species lineages. However, mitochondrial DNA (i.e., *co*b and *cox*1 genes) phylogenies produced five terminal branches resolving each species. When concatenated these combined sequences produced a robust phylogeny discriminating five distinct *Cladocopium* lineages associated with octocorals that were distinct and divergent from scleractinian endosymbionts (Figure 1a). Four of these new species lineages formed a monophyletic clade in the “C1‐radiation” (sensu Thornhill et al., 2014) that associate with hosts in the family Sarcophytidae (Figure 1b). The other distinct lineage grouped phylogenetically with the “C15‐radiation” and associated with the families Xeniidae and Lemnaliidae (Figure 1b). Maximum likelihood, parsimony, and Bayesian methodologies all produced similar results.

**FIGURE 2:**
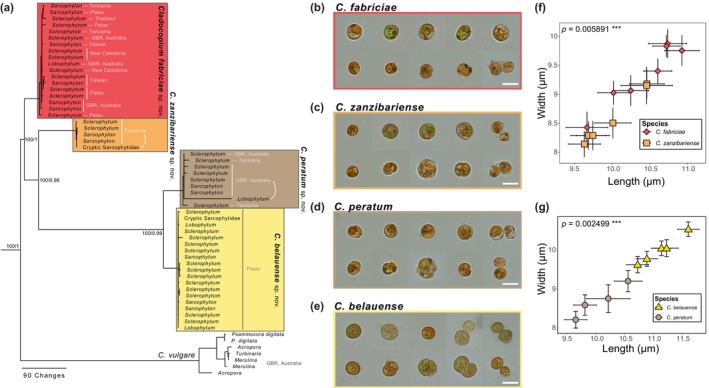
Phylogenetic and morphological data further resolving species of *Cladocopium* associated with octocorals in family Sarcophytidae in the C1‐radiation. (a) Maximum parsimony phylogeny of the *psb*A^ncr^ marker. Maximum likelihood bootstrap percentages based on 1000 replicates and posterior probability values are provided for nodes with ≥95% support. (b–e) Representative examples of cell images for each described species (scale bar = 10 μm), including (b) *C. fabriciae*; (c) *C. zanzibariense*; (d) *C. belauense*; and (e) *C. peratum*. (f) Variation in cell size means for *C. fabriciae* and *C. zanzibariense* from representative samples. (g) Variation in cell size means for *C. belauense* and *C. peratum* from representative samples. Error bars represent 95% confidence intervals.

**FIGURE 3:**
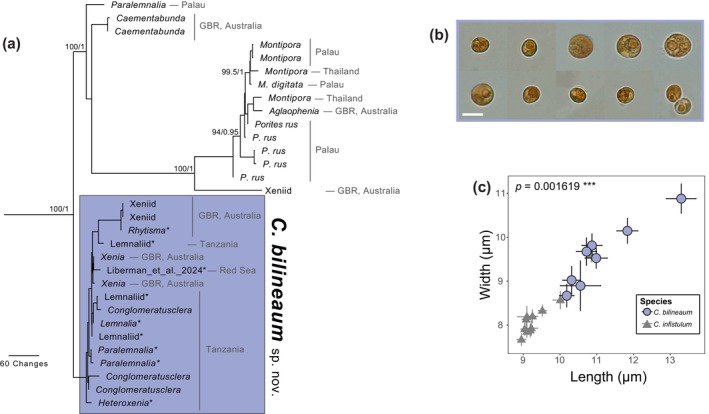
Phylogenetic and morphological data further resolving *Cladocopium bilineaum* associated with Xeniidae and Lemnaliidae in the C15‐radiation. (a) Maximum parsimony phylogeny of the *psb*A^ncr^. Values at nodes represent bootstrap support values from maximum likelihood analysis followed by posterior probability from MrBayes. Only nodes with ≥95% support are labeled. (b) Representative cell images of *C. bilineaum* (scale bar = 10 μm). (c) Variation in cell size means for *C. bilineaum* from representative samples compared to samples of *C. infistulum* from Lee et al. (2020). Error bars represent 95% confidence intervals.

The rapidly evolving *psb*A^ncr^ marker further discriminated inter‐ and intraspecific diversity. Subgeneric phylogenies generated from the *psb*A^ncr^ marker were congruent with the concatenated phylogeny based on conserved markers (Figures 2a and 3a). The high divergence of this marker between *Cladocopium* subclades precluded accurate alignments, and therefore, each is presented as an independent phylogeny. The inclusion of *psb*A^ncr^ marker sequences corresponding to formally described species from scleractinian hosts further justified formal species assignments to these evolutionarily independent lineages.

Several other genetic lineages were characterized representing additional species of *Cladocopium* spp. (not included here) as well as new species in the genera *Symbiodinium*, *Durusdinium*, and *Gerakladium* from the analyses of samples examined over the course of this study (data not shown). Because these occurred in and were represented by few samples, they were not included in the present formal taxonomic treatment.

### Morphology

Observations using light microscopy revealed no clear morphological features that discerned putative species. Mean cell sizes obtained from independent samples corresponding to each genetically defined lineage were statistically distinct when compared to closely related sibling species (Figures 2a,f,g and 3c). Multivariate analyses of variance (MANOVA; length and width as joint response variables) on cell‐size data from closely related species were significant for *Cladocopium fabriciae* (*n* = 8) versus *C. zanzibariense* (*n* = 6; Pillai's trace = 0.642, *F*_2,10_ = 8.96, *p* = 0.0059**; Figure 2b), *C. belauense* (*n* = 5) versus *C. peratum* (*n* = 4; Pillai's trace = 0.864, *F*_2,6_ = 19.11, *p* = 0.0025**; Figure 2c), and *C. bilineatum* versus the previously described *C. infistulum* (Lee et al., 2020; Pillai's trace = 0.577, *F*_2,16_ = 10.93, *p* = 0.0010**; Figure 3b).

### Zooxanthellae ccology: Host identity representing distinct habitats

**FIGURE 4:**
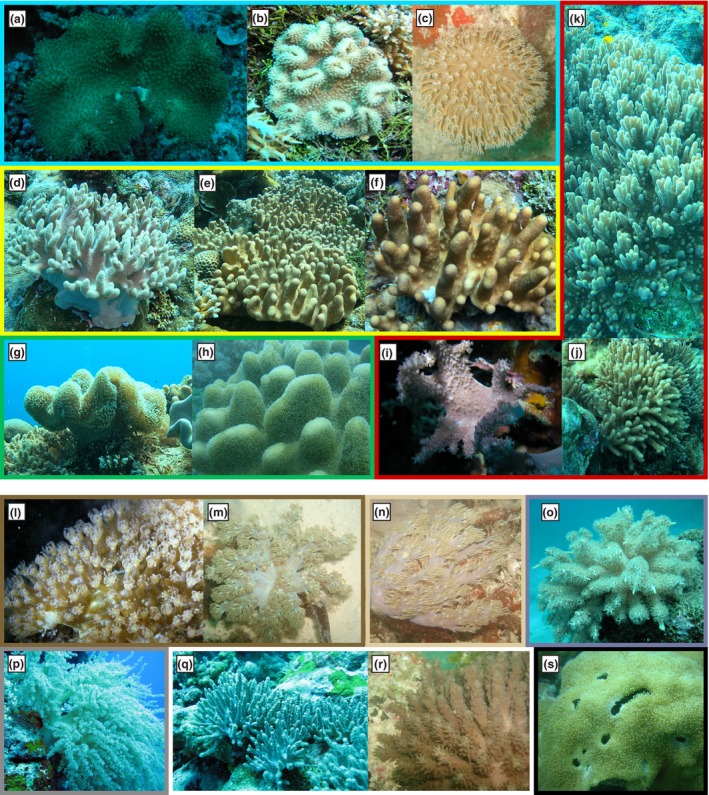
Images of the malacalcyonacean colonies surveyed in this study. Genera shown as follows: (a–c) *Sarcophyton* spp.; (d–f) *Lobophytum* spp.; (g–h) Colonies representative of a mixed clade in the family Sarcophytidae requiring taxonomic revision (see McFadden et al., 2006); (i–k) *Sclerophytum* spp.; (l, m) Colonies representative of unresolved genera in the family Xeniidae; (n) *Conglomeratusclera* sp.; (o) *Caementabunda* sp.; (p) *Lemnalia* sp.; (q, r) *Paralemnalia* spp.; (s) *Rhytisma* sp. Border colors and genus‐level identifications correspond to the host phylogeny shown in Figure S1.

Host identities were verified using the mitochondrial Mismatch Repair gene (*mtMutS*, also referred to as *msh*1) as used by McFadden et al. (2011). *Cladocopium fabriciae*, *C. peratum*, *C. belauense*, and *C. zanzibariense* were obtained from hosts in the family Sarcophytidae (Figures 1b and 4a), whereas *C. bilineaum* associated with members of families Xeniidae (including Heteroxenia, *Conglomeratusclera*, *Caementabunda*, and other genera named “cryptic Xeniidae” not fully resolved by the *mtMutS/msh*1 marker) and Lemnaliidae (*Rhytisma*, *Lemnalia*, and *Paralemnalia*; Figures 1b, 4l, Figure S1).

## DISCUSSION

The identification and characterization of octocoral‐associated dinoflagellates across Indo‐Pacific reef ecosystems revealed multiple independent species known to occur only in symbioses with octocorallian families Sarcophytidae, Xeniidae, and Lemnaliidae in the order Malacalcyonacea. Here we have satisfied the requirements of the evolutionary (genetic) and ecological (host genetics) species concepts with supporting morphological data. The biological species concept was satisfied through proxy, as multiple studies have shown equal scales of genetic resolution with the markers used here and microsatellite loci within the genus *Cladocopium* (Butler et al., 2023; Turnham et al., 2021).

Given the apparent ecological specialization of these lineages and estimates on the ages of modern *Cladocopium* spp., these mutualistic combinations evolved and have persisted for millions of years (LaJeunesse, 2005; LaJeunesse et al., 2018; Turnham et al., 2021). The octocoral symbionts described here represent constituents of two separate adaptive radiations (i.e., subclades) within the genus *Cladocopium* (Figure 1a; sensu Thornhill et al., 2014). Phylogenetically, four of these new species formed a well‐resolved clade separate from species specific to scleractinian stony corals in the C1 radiation (sensu Thornhill et al., 2014; Figure 1a), and the fifth species, *C. bilineaum*, was a separate lineage within the C15 radiation. These phylogenetic patterns further recognized symbiont lineages in the genus *Cladocopium* defined by their fidelity to specific host lineages (e.g., stony corals vs. soft corals), indicating the role of selection pressures corresponding to the host's intracellular habitat in driving much of this diversification (LaJeunesse, 2005; Thornhill et al., 2014).

### Octocoral host identity determines symbiont identity, above transmission mode

From the symbiont's perspective, the host‐habitat represents the most important resource from which it receives essential macro‐ and micronutrients and shelter for protection from consumers (e.g., filter‐feeders). Thus, any new attributes that may enhance the efficiency of each mutualistic partnership are likely preserved via natural selection. Eco‐evolutionary interactions between hosts and symbionts are strongly influenced by the way in which hosts acquire their symbionts from generation to generation. Vertical symbiont transmission facilitates symbiont speciation (Turnham et al., 2021), but the “host‐habitat” also influences the selection pressures that promote symbiont speciation via a process of ecological specialization (Schluter, 2009). The species defined here underscore how mutualistic interactions in hosts that horizontally acquire symbionts exert selection pressures that also promote symbiont diversification (e.g., Lewis et al., 2019).

Indo‐Pacific scleractinian corals with different modes of symbiont acquisition associate with different symbiont species. Horizontal transmitters display high fidelity for “host‐generalist” taxa (Butler et al., 2023), whereas vertical transmitters possess “host‐specialists” that exhibit specificity for a particular coral genus or species (LaJeunesse et al., 2014; Turnham et al., 2021; Wham et al., 2017). Although this same pattern appears to hold for most octocoral‐dinoflagellate mutualisms, *Cladocopium bilineaum* associated with groups of related hosts that display either horizontal and vertical transmission. In the family Xeniidae, both modes of transmission occur depending on the genus. Most xeniids are known to vertically transmit their endosymbionts during embryogenesis (Barneah et al., 2004), and *Heteroxenia* are known to obtain symbionts horizontally (Benayahu, 1991); yet this study and available ITS2 rDNA region sequence evidence indicated that they, too, associate with *C. bilineaum* (FitzPatrick et al., 2012; Goulet et al., 2008). *Cladocopium bilineaum* was also identified in representative samples of the family Lemnaliidae, which horizontally transmit their endosymbionts (Benayahu & Loya, 1983), and thus, *C. bilineaum* is an instance of a symbiont specific to both horizontally‐ and vertically‐transmitting anthozoan hosts. The regular association of both octocoral families with *C. bilineaum* over a large geographic area indicates the longevity and stability of these mutualisms (Figure 1d). This relationship with vertically and horizontally transmitting hosts leads to questions about the symbiont's origin and how heterogeneity in host transmission mode has affected its evolution.

The evolution of *Cladocopium bilineaum* and its specificity for both vertically and horizontally transmitting hosts with a shared evolutionary heritage suggests a stepwise expansion of its niche breadth. The approximate age of *C. bilineaum* is substantially younger than its hosts, considering that the oldest extant lineage in the genus *Cladocopium* evolved probably around 15 million years ago (MYA; Thornhill et al., 2014; LaJeunesse et al., 2018), whereas its hosts diverged from a common ancestor ~350–250 MYA (McFadden et al., 2021). One explanation is that this symbiont evolved while simultaneously associating with both host families; however, it may have specialized to one lineage before “jumping” to the other. For symbiont populations sequestered within a host's life cycle (i.e., vertical transmission), there has been a greater propensity to differentiate into new species through ecological isolation, genetic drift, and natural selection (e.g., LaJeunesse et al., 2014; Russell et al., 2019; Turnham et al., 2021). Nearly all vertically transmitting hosts studied thus far have possessed host‐specialized symbionts (LaJeunesse et al., 2014; Turnham et al., 2021; Wham et al., 2017). Given this, we propose that *C. bilineaum* initially speciated in vertically transmitting xeniids and, as a consequence, evolved compatibility with other horizontally transmitting xeniids and related Lemnaliidae.

Although the present study covered a broad host diversity, other zooxanthellate octocoral families common across the Indo‐Pacific, such as Briareidae, Cladiellidae, Clavulariidae, Isisidae, and Tubiporidae, were not examined in this study. Genetic evidence from past studies (see Barneah et al., 2004; Goulet et al., 2008; LaJeunesse et al., 2004) indicates that further research into these understudied groups will likely yield the description of new symbiont species uniquely in these hosts. Additionally, there is a multitude of additional host cnidarians as well as host taxa in the phyla Mollusca and Xenocoelomorpha (acoel flatworms) that possess zooxanthellae which warrant formal species descriptions.

### Widespread and regionally endemic species

Although geographic sampling remains incomplete, available evidence has indicated that each species has a distinct distribution range. *Cladocopium fabriciae* and *C. bilineaum* are geographically widespread, observed in hosts ranging from the western Indian Ocean to the central Pacific Ocean (Figure 1b,d). Previous studies appear to have also documented *C. bilineaum* in hosts from the Red Sea (Liberman et al., 2024), and therefore, it is reasonable to assume that *C. bilineaum* exists in association with xeniid and lemnaliid populations from the Red Sea to Tanzania and probably at locations further south along the eastern coast of Africa. Further sampling is required to substantiate this inference. In contrast to widespread species, *C. belauense* and *C. zanzibariense* occurred only in and dominated the Sarcophytidae host communities from Palau and Zanzibar, respectively (Figure 1b,c). Because they were not observed in hosts from any of the other sampling locations, these taxa appeared, and are characterized here as, regionally endemic.

The prevalence of regionally endemic *Cladocopium* spp. among octocoral (Sarcophytidae) communities in certain reef locations was notable. These biogeographic shifts in ecological dominance indicated that widely distributed generalists are not always the most competitive. Discerning biotic and abiotic factors that explain these regional differences in symbiont prevalence across host populations requires further consideration. Given that changes in symbiont dominance across reef habitats involve hosts with horizontal symbiont acquisition (LaJeunesse et al., 2004, 2010), external environments likely affect competition among host‐compatible symbionts, especially during the early establishment of these mutualisms (Coffroth et al., 2001). Coral reef ecosystems around the world differ substantially in mean temperature, irradiance, and nutrient availability, as well as in seasonal oscillations of these chemical–physical factors (e.g., Chollett et al., 2012). Presumably, any physiological differences between competing host‐generalist species would strongly dictate their relative ecological prevalence and abundance under different environmental settings (LaJeunesse et al., 2010).

The distribution and dispersal capabilities of widespread and endemic symbionts may have important implications for the resiliency of animal populations that are dependent on them, especially as oceans warm. Broad differences in biogeography have been well documented among formally described zooxanthellae species (Butler et al., 2023; LaJeunesse et al., 2014; Turnham et al., 2021). Unique distributions have often correlated to ecological attributes (light, heat, host range, etc.) specific to the niche of each species (e.g., LaJeunesse et al., 2014). Ultimately, the biogeographic descriptions of these new species are provisional, and additional sampling is needed from more reef systems (e.g., in eastern Africa, the central Indian Ocean, the Indonesian archipelago, etc.) across the Indo‐Pacific to resolve the full geographic range of each new species.

### Gaps in octocoral‐symbiont physiology

Physiological studies on octocorals and their symbionts are sparse and have often focused on the Atlantic Ocean (Pelosi et al., 2021; Ramsby et al., 2014; Rossi et al., 2018; Sammarco & Strychar, 2013; Steinberg et al., 2022; Strychar et al., 2005). Of the few bleaching surveys and thermal stress experiments published, pronounced differences in thermal tolerance among octocoral colonies were reported (Goulet et al., 2008; Sammarco & Strychar, 2013; Strychar et al., 2005). In these reports, rudimentary determination of the symbiont's identity constrained consideration of the symbiont's effect on colony thermal tolerance. When exposed to severe heat stress, xeniids are the most likely to bleach and die (Goulet et al., 2008). *Rhytisma*, however, which shares the same symbiont as the xeniids in this study (Figure 3a), has exhibited some of the highest thermal tolerances among octocorals. Until physiological characterizations are conducted on *Cladocopium bilineaum* and other Indo‐Pacific octocoral symbionts, we cannot know if they substantially influence thermal resilience among zooxanthellate octocorals as they do for stony corals (Aguilar et al., 2024; Berkelmans & van Oppen, 2006; Hoadley et al., 2019, 2021; Hume et al., 2015). Although differences in long‐standing regional environments (discussed above) and dispersal capabilities explain geographic patterns in ecological dominance, further physiological research may ultimately explain why certain sarcophytid symbionts are more abundant than others in a particular region.

## CONCLUSIONS

The formal characterization of Symbiodiniaceae diversity and integration of well‐defined species in physiological and ecological research remains ongoing. An established species taxonomy promotes the development and testing of hypotheses and thus drives new areas of research. What explains the wide distribution of *Cladocopium fabriciae* and *C. bilineaum*? What environmental parameters influence their relative dominance, and why are they displaced by certain endemic species? Does thermal tolerance influence patterns of distribution as has been observed with scleractinian symbionts? To what extent are populations representing each species connected? And how do different symbionts affect the relative growth and physiological resilience of octocorals, as observed for scleractinians? These and numerous other questions await future investigation and discovery.

Given previous estimates on the ages of genetically differentiated *Cladocopium* and their ecological niches (i.e., host specializations), these mutualistic relationships with octocorals likely evolved and have persisted for millions of years as was deduced for scleractinian‐specific species (i.e., adaptive radiations of host‐specialized taxa during the Pleistocene and Pliocene; LaJeunesse, 2005; LaJeunesse et al., 2018; Turnham et al., 2021). Ultimately, further consideration of what physiological traits, ecological processes, and environmental changes initiate the evolution of new species and then maintain these partnerships over geologic time‐scales is required; knowledge gained in this area also gives insight about future prospects of these zooxanthallate animals in a changing world.

## AUTHOR CONTRIBUTIONS

**Caleb C. Butler:** Conceptualization (lead); data curation (lead); formal analysis (equal); funding acquisition (supporting); investigation (equal); methodology (equal); project administration (lead); visualization (lead); writing – original draft (lead); writing – review and editing (equal). **Kira E. Turnham:** Investigation (supporting); methodology (supporting). **Andy Hess:** Investigation (supporting); methodology (supporting). **Todd C. LaJeunesse:** Conceptualization (equal); data curation (supporting); formal analysis (equal); funding acquisition (lead); investigation (equal); methodology (lead); project administration (equal); resources (lead); supervision (equal); validation (equal); visualization (equal); writing – original draft (equal); writing – review and editing (equal).

## Supporting information


**Figure S1.** Phylogeny of host octocorals used in this study with verified references in bold.

**Table S1.** List of *Cladocopium* samples with corresponding, ITS2 rDNA region type designation, host identity, area collected, and coordinates.

**Table S2.** List of primers used to amplify regions of both symbiont and host octocoral DNA.
