# Supplementary figures and images for: Resolving widespread and endemic dinoflagellates (Symbiodiniaceae) mutualistic with Indo‐Pacific octocorals reveals differences in specificity based on host phylogeny
Source: J Phycol. 2026 Feb 22;62(1):191–204. doi: 10.1111/jpy.70127 (PMC12961169; doi:10.1111/jpy.70127)

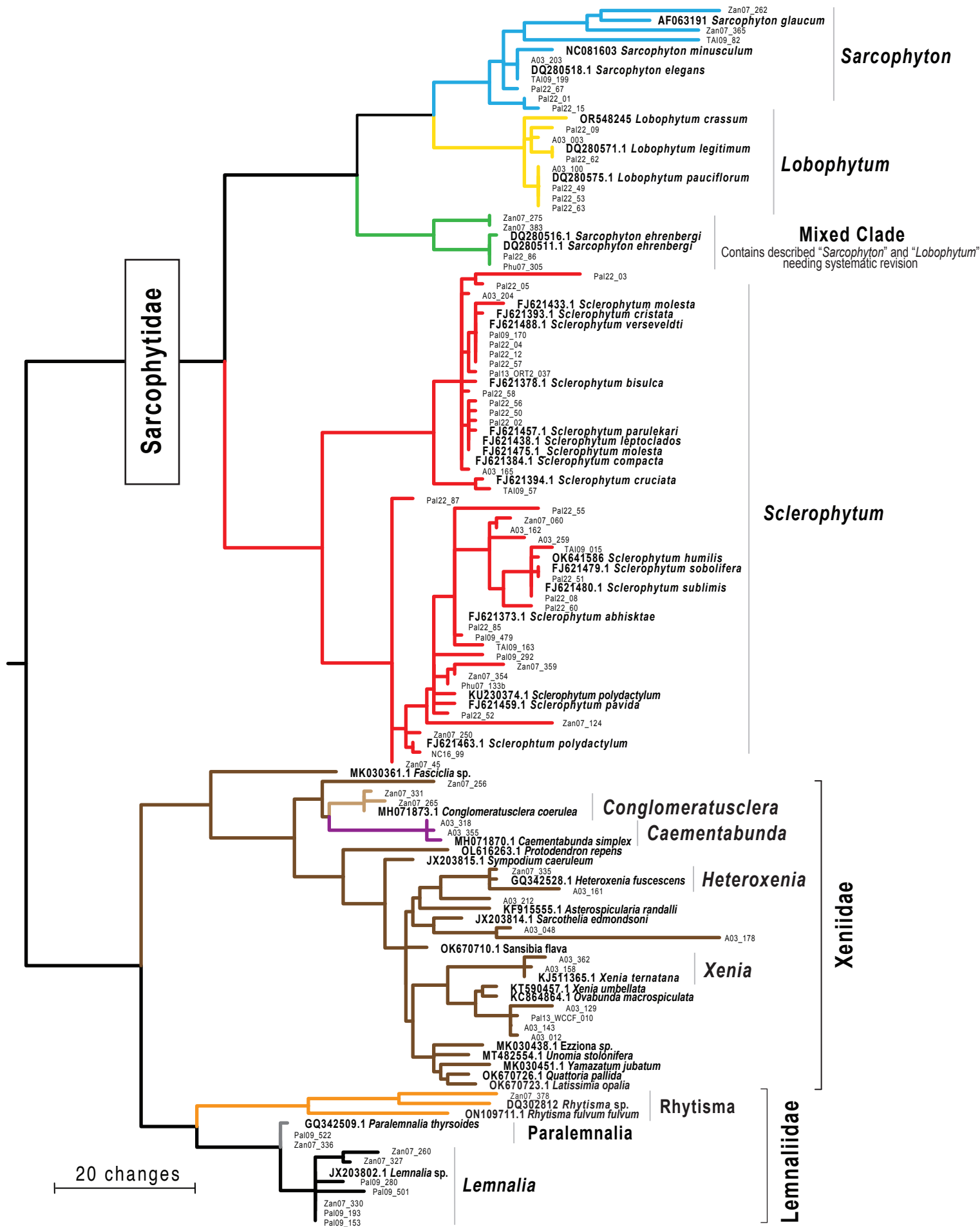

Supplement: Supplementary file 1 — Figure S1. Phylogeny of host octocorals used in this study with verified references in bold. [file JPY-62-191-s003.pdf]
