# Supplementary material for: Resolving widespread and endemic dinoflagellates (Symbiodiniaceae) mutualistic with Indo‐Pacific octocorals reveals differences in specificity based on host phylogeny
Source: J Phycol. 2026 Feb 22;62(1):191–204. doi: 10.1111/jpy.70127 (PMC12961169; doi:10.1111/jpy.70127)
Supplement: Supplementary file 2 — Table S1. List of Cladocopium samples with corresponding, ITS2 rDNA region type designation, host identity, area collected, and coordinates. [file JPY-62-191-s002.pdf]

Sample	Symbiont Species	ITS2 Type	Host Genus	Country	Site
A03_003	*C. fabriciae*	C1	*Lobophytum*	Australia	Feather Reef North
A03_005	*C. fabriciae*	C1	*Sarcophyton*	Australia	Feather Reef North
A03_012	*C. bilineaum*	C64	*Xenia*	Australia	Feather Reef North
A03_019	*C. peratum*	C65	*Lobophytum*	Australia	Feather Reef North
A03_035	Cryptic C1 radiation	C1	*Ifalukella*	Australia	Feather Reef North
A03_048	*C. bilineaum*	C64	*Xenia*	Australia	Feather Reef North
A03_049	*C. fabriciae*	C1	*Sarcophyton*	Australia	Feather Reef North
A03_064	Cryptic C15 radiation	C15		Australia	Feather Reef North
A03_072	*C. fabriciae*	C1	*Sarcophyton*	Australia	Feather Reef North
A03_076	*C. peratum*	C65		Australia	Feather Reef North
A03_100	*C. fabriciae*	C1	*Lobophytum*	Australia	Feather Reef North
A03_129	*C. bilineaum*	C64	*Xenia*	Australia	Feather Reef North
A03_131	*C. fabriciae*	C1	*Lobophytum*	Australia	Feather Reef North
A03_143	*C. bilineaum*	C64	*Xenia*	Australia	Feather Reef North
A03_149	Cryptic C1 radiation	C1	*Ifalukella*	Australia	Feather Reef South
A03_150	Cryptic C1 radiation	C1	*Anella*	Australia	Feather Reef South
A03_157	Cryptic C1 radiation	C1	*Astrogorgia*	Australia	Feather Reef South
A03_158	*C. bilineaum*	C64	*Xenia*	Australia	Feather Reef South
A03_159	Cryptic C15 radiation	C15		Australia	Feather Reef South
A03_161	*C. bilineaum*	C64	*Xenia*	Australia	Feather Reef South
A03_162	*C. peratum*	C65	*Sclerophytum*	Australia	Feather Reef South
A03_164	*C. bilineaum*	C64	*Xenia*	Australia	Feather Reef South
A03_165	*C. peratum*	C65	*Sclerophytum*	Australia	Feather Reef South
A03_175	*C. bilineaum*	C64	*Rhytisma*	Australia	Feather Reef South
A03_178	Cryptic C15 radiation	C15	*Xenia*	Australia	Feather Reef South
A03_202	Cryptic C1 radiation	C1	*Pinnigorgia*	Australia	Rib Reef North
A03_203	*C. peratum*	C65	*Sarcophyton*	Australia	Rib Reef North
A03_204	*C. peratum*	C65	*Sclerophytum*	Australia	Rib Reef North
A03_205	Cryptic C1 radiation	C1	*Astrogorgia*	Australia	Rib Reef North

A03_212	Cryptic C15 radiation	C15	*Xenia*	Australia	Rib Reef North
A03_241	*C. peratum*	C65	*Sclerophytum*	Australia	Rib Reef North
A03_242	*C. peratum*	C65	*Sarcophyton*	Australia	Rib Reef North
A03_250	Cryptic C1 radiation	C1	*Acabaria*	Australia	Rib Reef North
A03_259	*C. fabriciae*	C1	*Sclerophytum*	Australia	Rib Reef North
A03_306	Cryptic C3 radiation	C23	*Isis*	Australia	Rib Reef South
A03_308	Cryptic C3 radiation	C3	*Briareum*	Australia	Rib Reef South
A03_316	Cryptic C3 radiation	C23	*Isis*	Australia	Rib Reef South
A03_318	Cryptic C15 radiation	C15	*Xenia*	Australia	Rib Reef South
A03_355	Cryptic C15 radiation	C15	*Xenia*	Australia	Rib Reef South
A03_362	*C. bilineaum*	C64	*Xenia*	Australia	Curacao Island (MPN18-1805)
A03_394	*C. fabriciae*	C1	*Sarcophyton*	Australia	Curacao Island (MPN18-1805)
A03_055	*C. fabriciae*	C1	*Lobophytum*	Australia	Feather Reef North
NC16_132	*C. fabriciae*	C1	*Sclerophytum*	New Caledonia	Bouraké
NC16_133	*C. fabriciae*	C1	*Sclerophytum*	New Caledonia	Bouraké
NC16_134	*C. fabriciae*	C1	*Sclerophytum*	New Caledonia	Bouraké
NC16_84	*C. fabriciae*	C1	*Sarcophyton*	New Caledonia	Bouraké
NC16_93	*C. fabriciae*	C1	*Sclerophytum*	New Caledonia	Bouraké
NC16_97	*C. fabriciae*	C1	*Sclerophytum*	New Caledonia	Bouraké
NC16_99	*C. fabriciae*	C1	*Sclerophytum*	New Caledonia	Bouraké
Pal09_153	*C. bilineaum*	C64	*Rhytisma*	Palau	East Outer Reef
Pal09_170	*C. belauense*	C71	*Sclerophytum*	Palau	East Outer Reef
Pal09_179	*C. belauense*	C71	*Sarcophyton*	Palau	East Outer Reef
Pal09_193	*C. bilineaum*	C64	*Rhytisma*	Palau	East Outer Reef
Pal09_202	*C. belauense*	C71	*Sarcophyton*	Palau	East Outer Reef
Pal09_253	*C. belauense*	C71	*Lobophytum*	Palau	East Outer Reef
Pal09_257	*C. belauense*	C71	*Sarcophyton*	Palau	East Outer Reef
Pal09_280	*C. fabriciae*	C1	*Sclerophytum*	Palau	East Outer Reef
Pal09_292	*C. belauense*	C71	*Sclerophytum*	Palau	East Outer Reef
Pal09_322	*C. belauense*	C71	*Sclerophytum*	Palau	East Outer Reef

Pal09_474	*C. belauense*	C71	*Lobophytum*	Palau	West Reef
Pal09_479	*C. belauense*	C71	*Sclerophytum*	Palau	West Reef
Pal09_486	*C. belauense*	C71	*Sarcophyton*	Palau	West Reef
Pal09_501	*C. bilineaum*	C64	*Rhytisma*	Palau	West Reef
Pal09_509	*C. belauense*	C71	*Sarcophyton*	Palau	West Reef
Pal09_522	Cryptic C15 radiation	C15	*Rhytisma*	Palau	West Reef
Pal09_557	*C. belauense*	C71	*Lobophytum*	Palau	West Reef
Pal09_596	*C. fabriciae*	C1	*Sclerophytum*	Palau	West Reef
Pal13_ORT2_37	*C. belauense*	C71	*Sclerophytum*	Palau	Rebotel Reef
Pal13_WCCF_100	*C. fabriciae*	C1	*Sclerophytum*	Palau	West Channel Coral Flat
Pal22_01	*C. belauense*	C71	*Sarcophyton*	Palau	Rebotel Reef
Pal22_02	*C. belauense*	C71	*Sclerophytum*	Palau	Rebotel Reef
Pal22_03	*C. belauense*	C71	*Sclerophytum*	Palau	Rebotel Reef
Pal22_04	*C. belauense*	C71	*Sclerophytum*	Palau	Rebotel Reef
Pal22_05	*C. belauense*	C71	*Sclerophytum*	Palau	Rebotel Reef
Pal22_07	*C. belauense*	C71	*Sclerophytum*	Palau	Rebotel Reef
Pal22_08	*C. belauense*	C71	*Sclerophytum*	Palau	Rebotel Reef
Pal22_09	*C. belauense*	C71	*Lobophytum*	Palau	Rebotel Reef
Pal22_11	*C. fabriciae*	C1		Palau	Rebotel Reef
Pal22_12	*C. belauense*	C71	*Sclerophytum*	Palau	Rebotel Reef
Pal22_13	*C. belauense*	C71		Palau	Rebotel Reef
Pal22_15	*C. belauense*	C71	*Sarcophyton*	Palau	Rebotel Reef
Pal22_49	*C. belauense*	C71	*Lobophytum*	Palau	Rebotel Reef
Pal22_50	*C. belauense*	C71	*Sclerophytum*	Palau	Rebotel Reef
Pal22_51	*C. belauense*	C71	*Sclerophytum*	Palau	Rebotel Reef
Pal22_52	*C. fabriciae*	C1	*Sclerophytum*	Palau	Rebotel Reef
Pal22_53	*C. belauense*	C71	*Lobophytum*	Palau	Rebotel Reef
Pal22_55	*C. belauense*	C71	*Sclerophytum*	Palau	Rebotel Reef
Pal22_56	*C. belauense*	C71	*Sclerophytum*	Palau	Rebotel Reef
Pal22_57	*C. belauense*	C71	*Sclerophytum*	Palau	Rebotel Reef

Pal22_58	*C. belauense*	C71	*Sclerophytum*	Palau	Rebotel Reef
Pal22_59	*C. belauense*	C71	*Sclerophytum*	Palau	Rebotel Reef
Pal22_60	*C. belauense*	C71	*Sclerophytum*	Palau	Rebotel Reef
Pal22_62	*C. belauense*	C71	*Lobophytum*	Palau	Rebotel Reef
Pal22_63	*C. belauense*	C71	*Lobophytum*	Palau	Rebotel Reef
Pal22_66	*C. fabriciae*	C1	*Sclerophytum*	Palau	Rebotel Reef
Pal22_67	*C. belauense*	C71	*Sarcophyton*	Palau	Rebotel Reef
Pal22_83	*C. fabriciae*	C1	*Sclerophytum*	Palau	Rebotel Reef
Pal22_84	*C. fabriciae*	C1	*Sclerophytum*	Palau	West Channel Coral Flat
Pal22_85	*C. fabriciae*	C1	*Sclerophytum*	Palau	West Channel Coral Flat
Pal22_86	*C. belauense*	C71	Cryptic Sarcophytidae	Palau	West Channel Coral Flat
Pal22_90	*C. belauense*	C71	*Sclerophytum*	Palau	West Channel Coral Flat
Pal22_91	*C. fabriciae*	C1	*Sclerophytum*	Palau	West Channel Coral Flat
Phu07_133b	*C. fabriciae*	C1	*Sclerophytum*	Thailand	Phiphi Lae
Phu07_175	*C. fabriciae*	C1	*Sclerophytum*	Thailand	Phiphi Lae
Phu07_196	Cryptic C3 radiation	C3	*Dendronepthya*	Thailand	Racha
Phu07_305	*C. fabriciae*	C1	*Sarcophyton*	Thailand	Hae
TAI09_015	*C. fabriciae*	C1	*Sclerophytum*	Taiwan	Power Plant
TAI09_057	*C. fabriciae*	C1	*Sclerophytum*	Taiwan	Power Plant
TAI09_073	*C. fabriciae*	C1	*Lobophytum*	Taiwan	Power Plant
TAI09_082	*C. fabriciae*	C1	*Sarcophyton*	Taiwan	Power Plant
TAI09_085	*C. fabriciae*	C1	*Sclerophytum*	Taiwan	Power Plant
TAI09_088	*C. fabriciae*	C1	*Sarcophyton*	Taiwan	Power Plant
TAI09_090	*C. fabriciae*	C1	*Xenia*	Taiwan	Power Plant
TAI09_141	*C. fabriciae*	C1	*Lobophytum*	Taiwan	Power Plant
TAI09_163	*C. fabriciae*	C1	*Sclerophytum*	Taiwan	Power Plant
TAI09_199	*C. fabriciae*	C1	*Sclerophytum*	Taiwan	Power Plant
Zan07_009	*C. fabriciae*	C1r	*Sarcophyton*	Zanzibar, Tanzania	Changuu
Zan07_026	*C. fabriciae*	C1r	*Sclerophytum*	Zanzibar, Tanzania	Changuu
Zan07_045	*C. fabriciae*	C1r	*Sclerophytum*	Zanzibar, Tanzania	Changuu

Zan07_060	*C. zanzibariense*	C107ab	*Sclerophytum*	Zanzibar, Tanzania	Changuu
Zan07_124	*C. peratum*	C65	*Sclerophytum*	Zanzibar, Tanzania	Changuu
Zan07_173	*C. fabriciae*	C1r	*Sarcophyton*	Zanzibar, Tanzania	Changuu
Zan07_250	*C. zanzibariense*	C107ab	*Sclerophytum*	Zanzibar, Tanzania	Bawe
Zan07_256	*C. bilineaum*	C64	*Xenia*	Zanzibar, Tanzania	Banda Kuu
Zan07_260	*C. bilineaum*	C64	*Rhytisma*	Zanzibar, Tanzania	Banda Kuu
Zan07_261	*C. bilineaum*	C64	*Rhytisma*	Zanzibar, Tanzania	Banda Kuu
Zan07_262	*C. zanzibariense*	C107ab	*Sarcophyton*	Zanzibar, Tanzania	Banda Kuu
Zan07_263	*C. fabriciae*	C1r	*Sclerophytum*	Zanzibar, Tanzania	Banda Kuu
Zan07_265	*C. bilineaum*	C64	*Caementabunda*	Zanzibar, Tanzania	Banda Kuu
Zan07_275	*C. zanzibariense*	C107ab	*Sarcophyton*	Zanzibar, Tanzania	Banda Kuu
Zan07_297	*C. bilineaum*	C64	*Rhytisma*	Zanzibar, Tanzania	Banda Kuu
Zan07_327	*C. bilineaum*	C64	*Rhytisma*	Zanzibar, Tanzania	Banda Kuu
Zan07_328	*C. zanzibariense*	C107ab	*Sarcophyton*	Zanzibar, Tanzania	Banda Kuu
Zan07_330	*C. bilineaum*	C64	*Rhytisma*	Zanzibar, Tanzania	Banda Kuu
Zan07_331	*C. bilineaum*	C64	*Xenia*	Zanzibar, Tanzania	Banda Kuu
Zan07_335	*C. bilineaum*	C64	*Xenia*	Zanzibar, Tanzania	Banda Kuu
Zan07_336	*C. bilineaum*	C64	*Rhytisma*	Zanzibar, Tanzania	Banda Kuu
Zan07_359	*C. peratum*	C65	*Sclerophytum*	Zanzibar, Tanzania	Banda Kuu
Zan07_365	*C. zanzibariense*	C107ab	*Sarcophyton*	Zanzibar, Tanzania	Banda Kuu
Zan07_378	*C. bilineaum*	C64	*Rhytisma*	Zanzibar, Tanzania	Banda Kuu
Zan07_383	*C. peratum*	C65	Cryptic Sarcophytidae	Zanzibar, Tanzania	Banda Kuu

Coordinates
-17.534018, 146.386874
-17.534018, 146.386874
-17.534018, 146.386874
-17.534018, 146.386874
-17.534018, 146.386874
-17.534018, 146.386874
-17.534018, 146.386874
-17.534018, 146.386874
-17.534018, 146.386874
-17.534018, 146.386874
-17.534018, 146.386874
-17.534018, 146.386874
-17.534018, 146.386874
-17.534018, 146.386874
-17.534018, 146.386874
-17.534018, 146.386874
-17.534018, 146.386874
-17.534018, 146.386874
-17.534018, 146.386874
-17.534018, 146.386874
-17.534018, 146.386874
-17.534018, 146.386874
-17.534018, 146.386874
-17.534018, 146.386874
-17.534018, 146.386874
-18.491545, 146.871934
-18.491545, 146.871934
-18.491545, 146.871934
-18.491545, 146.871934

-18.491545, 146.871934
-18.491545, 146.871934
-18.491545, 146.871934
-18.491545, 146.871934
-18.491545, 146.871934
-18.491545, 146.871934
-18.491545, 146.871934
-18.491545, 146.871934
-18.491545, 146.871934
-18.491545, 146.871934
-18.665308, 146.565102
-18.665308, 146.565102
-17.534018, 146.386874
-21.972243, 165.971871
-21.972243, 165.971872
-21.972243, 165.971873
-21.972243, 165.971874
-21.972243, 165.971875
-21.972243, 165.971876
-21.972243, 165.971877
7.262653; 134.519233
7.262653; 134.519233
7.262653; 134.519233
7.262653; 134.519233
7.262653; 134.519233
7.262653; 134.519233
7.262653; 134.519233
7.262653; 134.519233
7.262653; 134.519233
7.262653; 134.519233

7.544778, 134.468417
7.544778, 134.468418
7.544778, 134.468419
7.544778, 134.468420
7.544778, 134.468421
7.544778, 134.468422
7.544778, 134.468423
7.544778, 134.468424
7.253508, 134.219031
7.544778, 134.468417
7.253508, 134.219031
7.253508, 134.219031
7.253508, 134.219031
7.253508, 134.219031
7.253508, 134.219031
7.253508, 134.219031
7.253508, 134.219031
7.253508, 134.219031
7.253508, 134.219031
7.253508, 134.219031
7.253508, 134.219031
7.253508, 134.219031
7.253508, 134.219031
7.253508, 134.219031
7.253508, 134.219031
7.253508, 134.219031
7.253508, 134.219031
7.253508, 134.219031
7.253508, 134.219031
7.253508, 134.219031

7.253508, 134.219031
7.253508, 134.219031
7.253508, 134.219031
7.253508, 134.219031
7.253508, 134.219031
7.253508, 134.219031
7.253508, 134.219031
7.253508, 134.219031
7.544778, 134.468417
7.544778, 134.468418
7.544778, 134.468419
7.544778, 134.468420
7.544778, 134.468421
7.701223, 98.769896
7.701223, 98.769896
7.578656, 98.355800
7.701223, 98.769896
21.953111, 120.754722
21.953111, 120.754723
21.953111, 120.754724
21.953111, 120.754725
21.953111, 120.754726
21.953111, 120.754727
21.953111, 120.754728
21.953111, 120.754729
21.953111, 120.754730
21.953111, 120.754731
-6.120110, 39.168786
-6.120110, 39.168786
-6.120110, 39.168786

-6.120110, 39.168786
-6.120110, 39.168786
-6.120110, 39.168786
-6.15475, 39.126583
-5.716666, 39.297771
-5.716666, 39.297771
-5.716666, 39.297771
-5.716666, 39.297771
-5.716666, 39.297771
-5.716666, 39.297771
-5.716666, 39.297771
-5.716666, 39.297771
-5.716666, 39.297771
-5.716666, 39.297771
-5.716666, 39.297771
-5.716666, 39.297771
-5.716666, 39.297771
-5.716666, 39.297771
-5.716666, 39.297771
-5.716666, 39.297771
-5.716666, 39.297771
-5.716666, 39.297771
