# Supplementary material for: Resolving widespread and endemic dinoflagellates (Symbiodiniaceae) mutualistic with Indo‐Pacific octocorals reveals differences in specificity based on host phylogeny
Source: J Phycol. 2026 Feb 22;62(1):191–204. doi: 10.1111/jpy.70127 (PMC12961169; doi:10.1111/jpy.70127)
Supplement: Supplementary file 3 — Table S2. List of primers used to amplify regions of both symbiont and host octocoral DNA. [file JPY-62-191-s001.pdf]

Table S2: Primers used to amplify regions of both symbiont and host octocoral DNA.

**Genetic Marker**	**Primers**	**Fragment size**	**Reference**
DI/D2 LSU rDNA	28S-F 5'-CCCGCTGAATTTAAGCATATAAGTAAGCGG-3'	629-650bp	Zardoya et al. 1995
	28S-R 5'-GTTAGACTCCTTGGTCCGTGTTTCAAGA-3		
cox 1	Dinocox1F: 5′-AAAAATTGTAATCATAAACGCTTAGG-3′	971bp	Zhang et al. 2008
	Dinocox1R: 5′-TGTTGAGCCACCTATAGTAAACATTA-3′		
cob	Dinocob1F 5'-ATGAAATCTCATTTACAWWCATATCCTTGTCC-3'	896bp	Zhang et al. 2008
	Dinocob1R 5'-TCTCTTGAGGKAATTGWKMACCTATCCA-3'		
cp23s rDNA	23S1 5'-GGCTGTAACTATAACGGTCC-3'	586bp	Zhang et al. 2000
	23S2 5'- CCATCGTATTGA ACCCAGC-3'		
psb Ancr	7.4-Forw 5'- GCATGAAAGAAATGCACACAACTTCCC –3'	1068-1421bp	Moore et al. 2003
	7.8-Rev 5'- GGTTCTCTTATTCCATCAATATCTACTG -3'		
msh1/mMutS	ND42599F 5'-GCCATTATGGTTAACTATTAC-3'	801-849bp	France & Hoover 2002
	MUT3458R 5'-TSGAGCAAAAGCCACTCC-3'		Sánchez et al. 2003

REFERENCES

Frances, S.C., Hoover, L.L. (2002). DNA sequences of the mitochondrial COI gene have low levels of divergence among deep-sea octocorals (Cnidaria: Anthozoa). *Hydrobiologia 471,* 149–155. https://doi.org/10.1023/A:1016517724749.

Moore, R. B., Ferguson, K. M., Loh, W. K., Hoegh-Guldberg, O., & Carter, D. A. (2003). Highly organized structure in the non-coding region of the psbA minicircle from clade C *Symbiodinium* . *International Journal of Systematic and Evolutionary Microbiology* , *53* (6), 1725-1734. https://doi.org/10.1099/ijs.0.02594-0

Sánchez, J. A., & McFadden, C. S. Frances S. C., Lasker H. R. (2003). Molecular phylogenetic analyses of shallow-water Caribbean octocorals. *Marine Biology* , 142:975-987. https://doi.org/10.1007/s00227-003-1018-7

Zardoya, R., Castas, E., & Lopez-Rodas, V., Garrido-Pertierra, A., Bautista, J.M. (1995). Revised dinoflagellate phylogeny inferred from molecular analysis of large-subunit ribosomal RNA gene sequences. *Journal of Molecular Evolution* , *41* (5). https://doi.org/10.1007/BF00175822

Zhang, Z., Green, B. R., & Cavalier-Smith, T. (2000). Phylogeny of ultra-rapidly evolving dinoflagellate chloroplast genes: A possible common origin for sporozoan and dinoflagellate plastids. *Journal of Molecular Evolution* , 51(1), 26–402. https://doi.org/10.1007/s002390010064

Zhang, H., Bhattacharya, D., Maranda, L., & Lin, S. (2008). Mitochondrial cob and cox1 genes and editing of the corresponding mRNAs in Dinophysis acuminata from Narragansett Bay, with special reference to the phylogenetic position of the genus Dinophysis. *Applied and Environmental Microbiology* , 74(5), 1546–1554. https://doi.org/10.1128/AEM.02103-07
